# The Bacteriological World

**Published:** 1891-01

**Authors:** 


THE BACTERIOLOGICAL WORLD is a new product of the commencing year, and will issue 5,000 copies this month. The first number will contain the following articles: Frontispiece, Pasteur's and Koch's pictures ; Study of Bacteriology, preface, introduction, etc.; Lesson First, Generalities on Germs, Spontaneous Generation ; Original articles, Actinomycosis in Man and Beast (big jaw of cattle.) Foreign and home investigations ; Surgical Bacteriology, Bacterial Complication of Wounds (Ogston, Rosenbach, Cornil, Babès, etc.); Medical Bacteriology, Immunity, by Dr. Bouchard, Paris, France; Hygienic Bacteriology, Hydrophobia, by Dr. Paul Gibier, Pasteur's Institute, New York City ; True and Spurious Bovine Vaccination and Complications, by Paul Evans, Pathological Laboratory, Missouri Agricultural Experiment Station; Clinical Notes, Pambotano in Malaria (a specific substitute for quinine), Tarro-Petrolene (or petrolene compound), Pyoktanine ; Editorial, Koch's Treatment of Tuberculosis ; Notes from Laboratories, Pasteur's laboratory and others. It is edited by Paul Paquin, M. D., Columbia, Mo.

